# Improved Viability of Probiotics via Microencapsulation in Whey-Protein-Isolate-Octenyl-Succinic-Anhydride-Starch-Complex Coacervates

**DOI:** 10.3390/molecules28155732

**Published:** 2023-07-28

**Authors:** Qingqing Liu, Chutian Lin, Xue Yang, Shuwen Wang, Yunting Yang, Yanting Liu, Mingming Xiong, Yisha Xie, Qingbin Bao, Yongjun Yuan

**Affiliations:** 1 Chongqing Key Laboratory of Speciality Food Co-Built by Sichuan and Chongqing, School of Food and Bioengineering, Xihua University, Chengdu 610039, China; liuqing_861006@163.com (Q.L.);; 2 Key Laboratory of Grain and Oil Processing and Food Safety of Sichuan Province, College of Food and Bioengineering, Xihua University, Chengdu 610039, China

**Keywords:** probiotic, microencapsulation, whey-protein isolate, octenyl-succinic-anhydride starch, complex coacervation

## Abstract

The aim of this study was to microencapsulate probiotic bacteria (*Lactobacillus acidophilus* 11073) using whey-protein-isolate (WPI)–octenyl-succinic-anhydride-starch (OSA-starch)-complex coacervates and to investigate the effects on probiotic bacterial viability during spray drying, simulated gastrointestinal digestion, thermal treatment and long-term storage. The optimum mixing ratio and pH for the preparation of WPI-OSA-starch-complex coacervates were determined to be 2:1 and 4.0, respectively. The combination of WPI and OSA starch under these conditions produced microcapsules with smoother surfaces and more compact structures than WPI-OSA starch alone, due to the electrostatic attraction between WPI and OSA starch. As a result, WPI-OSA-starch microcapsules showed significantly (*p* < 0.05) higher viability (95.94 ± 1.64%) after spray drying and significantly (*p* < 0.05) better protection during simulated gastrointestinal digestion, heating (65 °C/30 min and 75 °C/10 min) and storage (4/25 °C for 12 weeks) than WPI-OSA-starch microcapsules. These results demonstrated that WPI-OSA-starch-complex coacervates have excellent potential as a novel wall material for probiotic microencapsulation.

## 1. Introduction

Probiotics are living microorganisms that can provide a lot of benefits to the health of a host when properly ingested [1,2]. Many probiotics, including *Bifidobacterium*, *Lactobacillus* and others, have been widely used in functional foods, beverages and so on [1,3]. However, the instability of probiotics during food processing, human gastrointestinal digestion, long-term storage and more is still a great challenge [4,5]. Microencapsulation is considered to be an effective method to provide physical protection, and thereby increase the viability of probiotics [6]. Among the various microencapsulation methods, spray drying is considered to be one of the most widely used methods due to its moderate price, ease of operation, continuous production and excellent safety [7].

It is universally acknowledged that the choice of wall materials is of great importance for microencapsulation [8]. For the microencapsulation of probiotics, proteins and polysaccharides are the most commonly used wall materials [9]. Proteins have good film-forming properties, but are easily degraded by pepsin in gastric fluids and have a tendency to aggregate [10]. Polysaccharides can maintain their stability in the stomach due to their good resistance to acid and pepsin, which can improve the compactness of the microcapsules and be beneficial for the targeted release of probiotics. On the other hand, polysaccharides generally cannot provide adequate protection for probiotics due to their poor film-forming properties and the large pores between their molecular chains [11]. Therefore, compared to protein/polysaccharide alone, the probiotic microcapsules with higher encapsulation viability and more stability could be prepared by the combination of them [10,11].

Whey-protein isolate (WPI), isolated from cheese-making waste, is an attractive encapsulant as it has been found to be effective in protecting probiotic bacteria during spray drying [12]. However, WPI cannot provide an effective moisture barrier and therefore cannot effectively protect probiotics during storage due to its hydrophilic nature [12,13]. Octenyl succinic anhydride (OSA) starch, i.e., starch modified with OSA groups (an esterifying agent), has been recognised as a safe polysaccharide by the United States Food and Drug Administration (FDA) [7,14]. The hydrophobicity of OSA starch is relatively high with a contact angle of about 80° due to the presence of lipophilic octenyl groups [15,16]. In addition, the digestibility of OSA starch was relatively low due to the spatial obstruction caused by the OSA groups, which reduces its contact with digestive enzymes [17]. According to Wang et al., the relatively high hydrophobicity (contact angle of 76.10°) and digestion resistance of modified starch (propionylated potato starch) was beneficial in protecting probiotics during spray drying, simulated gastrointestinal (SGI) digestion and storage [18]. OSA starch alone cannot provide sufficient protection for probiotics during spray drying. As reported by Cruz-Benítez et al. [19], the viability of *Lactobacillus pentosus* in OSA starch microcapsules after spray drying was only about 65%. The weak protective effect of the OSA starch microcapsules could be attributed to the poor solubility and film-forming property of OSA starch, resulting in an uneven film formation at the beginning of the drying process and therefore the shrunken and wrinkled surfaces of the spray-dried microcapsules [19,20].

Complex coacervation is one of the most commonly used methods for combining protein with polysaccharide, which occurs as a phase separation of the water solution containing them with opposite electrical charges and the formation of a coacervate layer surrounding the bioactive compound [21]. Probiotic microcapsules formed via complex coacervation have advantages in encapsulation viability, protective capability, controlled release, etc. [9]. To date, complex coacervates of pea-protein isolate–sugar-beet pectin, sodium caseinate–sugar-beet pectin, soy-protein isolate–pectin, soy-protein isolate–peach-gum polysaccharide, WPI–gum arabic, soy-protein isolate–carrageenan, casein–chitosan and others have been used for probiotic microencapsulation [9,10,21,22,23]. However, the use of WPI-OSA-starch-complex coacervates in probiotic microencapsulation has not been reported. Therefore, in the present study, WPI-OSA-starch-complex coacervates were used as the wall material for *Lactobacillus acidophilus* encapsulation via spray drying. The SGI digestion, thermal resistance and storage properties of microencapsulated and free probiotic bacteria and the physicochemical properties and microstructure of probiotic microcapsules were further investigated.

## 2. Results and Discussion

### 2.1. Formation of WPI-OSA-Starch-Complex Coacervates

#### 2.1.1. Turbidity

Complex coacervation of charged protein and polysaccharide can be reflected by the change in turbidity of the solution at the macroscopic level. Therefore, turbidity titration has usually been used to investigate the whole process of complex coacervation and to measure the conditions under which the maximum complex coacervates can be formed [24].

**Figure 1:**
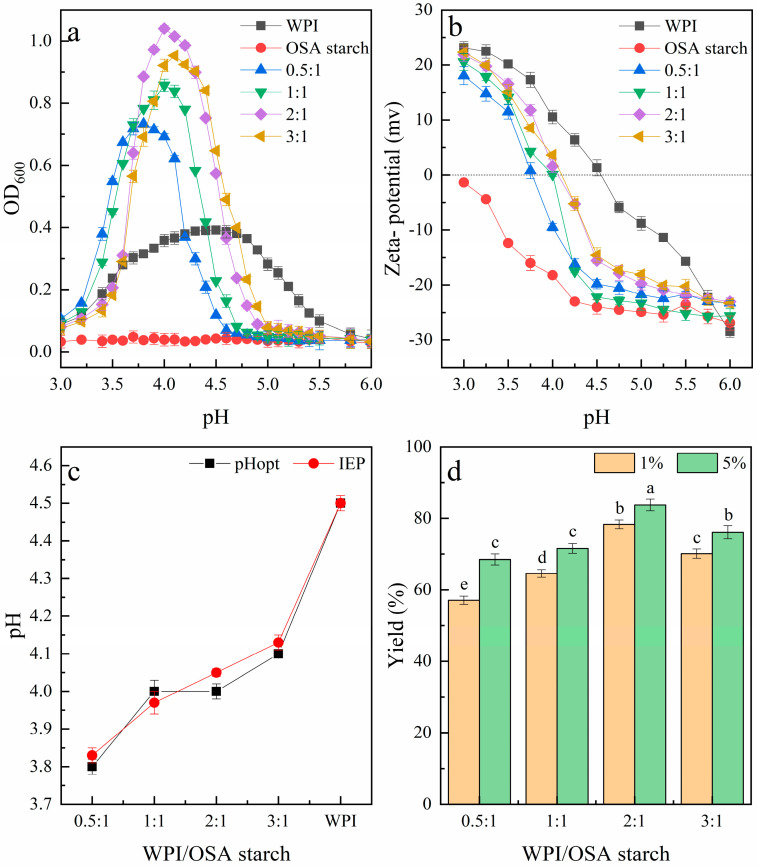
Effects of mixing ratio, pH and total polymer concentration on the formation of WPI-OSA-starch-complex coacervates. (**a**) Zeta potential and (**b**) turbidity of WPI-OSA starch alone and WPI-OSA starch mixtures at different ratios as a function of pH; (**c**) the pHopt and IEP of WPI-OSA-starch mixtures at different ratios; (**d**) the yield of WPI-OSA-starch-complex coacervates at different ratios and different total polymer concentrations. Different lowercase letters in (**d**) mean significant differences (*p* < 0.05).

As shown in Figure 1a, the turbidity of the WPI-OSA-starch mixtures increased firstly and then decreased as the pH lowered from 2.0 to 6.5. The pHopt was determined to be 3.8, 3.9, 4.0 and 4.1 when the mixing ratios were 0.5:1, 1:1, 2:1 and 3:1, respectively. These results indicated that an increase in WPI in the mixture required an increase in the pHopt, similar to the report of Qiu et al. [25] and Tian et al. [26]. In addition, the maximum turbidity value was significantly influenced by the mixing ratio. As the mixing ratio of WPI-OSA starch was 0.5:1, i.e., the amount of WPI was lower than that of OSA starch, only weak interactions occurred between WPI and OSA starch, resulting in relatively low turbidity. Similar phenomena have also been reported for other combinations of protein polysaccharide, such as hemp-protein isolate–gum arabic, gelatin–OSA-modified kudzu starch and others [27,28]. The reason for this can be attributed to there being too many anionic groups in the polysaccharide, resulting in strong electrostatic repulsion between them. As the mixing ratio increased, the turbidity first increased and then decreased. The highest turbidity was observed when the WPI-OSA starch ratio was 2:1 and the pH was 4.0, indicating the formation of WPI-OSA-starch complex coacervates with maximum insolubility and stability. Similar phenomena were also observed by Plati et al. [27] and Tian et al. [26]. Thus, this is the possible optimum mixing ratio for the correct saturation of the charged sites of the OSA with WPI molecules [27]. Turbidity decreased when the ratio was further increased to 3:1; this was also in agreement with the report of Plati et al. [27] and Tian et al. [26] and could be attributed to an excess of WPI in the solution, which could not bind to the OSA starch, being replaced by protein self-aggregation [27]. This assumption was supported by a shift of pHopt closer to the PI of WPI.

#### 2.1.2. Zeta Potential

As the complex coacervation of protein and polysaccharide is mainly motivated by electrostatic attraction, their zeta potential could provide important information about the interaction between them, as well as the formation and stability of complex coacervates [29].

As shown in Figure 1b, the zeta potential of the WPI decreased with increasing pH from 3.0 to 6.0 and changed from positive to negative at about pH 4.5, which is its IEP, similar to the previous report [30]. In contrast, OSA starch mainly maintained a negative charge and decreased with increasing pH from 3.0 to 6.0, which is similar to the previous report of Zhao et al. [28]. The zeta potential of all the WPI-OSA-starch blends was intermediate between the two single biopolymers. The IEP was found to be 3.83 at a mixing ratio of 0.5:1 (WPI-OSA starch). When the mixing ratio of WPI-OSA starch was increased from 0.5:1 to 3:1, the IEP values also increased, reaching a maximum of 4.13 at a mixing ratio of 3:1 (Figure 1b). Similar phenomena have been observed previously for other combinations of protein polysaccharide, e.g., hemp-protein isolate–gum arabic, pea-protein isolate–beet pectin and others [27,29].

#### 2.1.3. Comparison of IEP and pHopt

As shown in Figure 1c, the pHopt determined via turbidimetric analysis at each mixing ratio approximately coincided with the IEP. This phenomenon is consistent with the previous reports that the maximum turbidity of the mixed solutions of protein polysaccharide is generally observed at their IEP [27,31,32], confirming again that electrostatic interaction is the main driving force for the complex coacervation.

#### 2.1.4. Yield of Complex Coacervates

As shown in Figure 1d, at a total polymer concentration of 1%, the yield of the complex coacervates increased with increasing WPI-OSA-starch-mixing ratio and the highest amount of complex coacervates (78.33 ± 2.24%) was formed when the WPI-OSA-starch-mixing ratio was 2:1, which corresponded to the highest turbidity. A similar yield of complex coacervates has also been obtained for other systems, such as WPI–quince-seed mucilage, soybean-protein isolate–soluble dietary fiber of Sichuan pepper seed and others [31,33]. However, as the mixing ratio was further increased, the yield of the complex coacervates started to decrease, which was attributed to the fact that the excess WPI was unable to bind with the OSA starch and remained soluble in the equilibrium phase [34]. In addition, the yield of WPI-OSA-starch-complex coacervates increased to 83.73 ± 2.75% as the total biopolymer concentration increased to 5%, similar to the report of Bhargavi et al. [35]. The reason for this can be attributed to the increased number of charged sites available for electrostatic interaction at a high biopolymer concentration [35].

Therefore, the conditions for the preparation of WPI-OSA-starch-complex coacervates for probiotic encapsulation were selected as follows: mixing ratio 2:1, pH 4.0, total polymer concentration of 5%.

### 2.2. FTIR Spectroscopy

**Figure 2:**
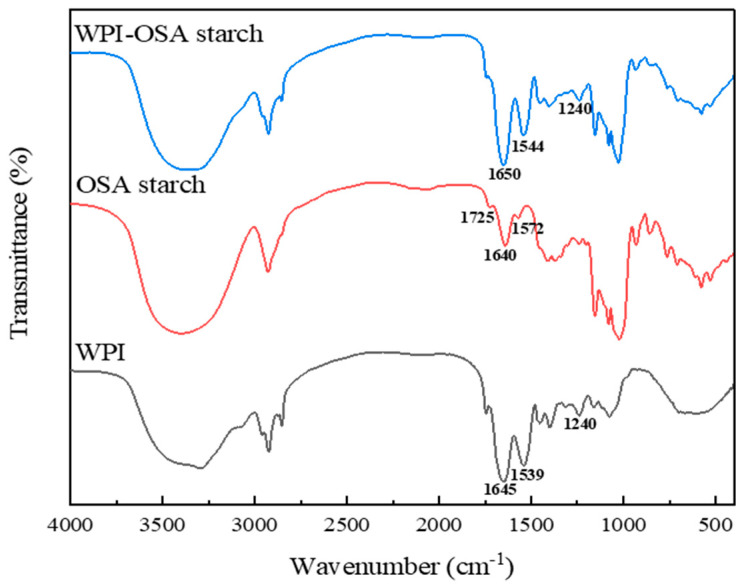
FTIR spectrogram of WPI microcapsules, OSA-starch microcapsules and WPI-OSA-starch microcapsules.

Figure 2 shows the FTIR spectroscopy of probiotic microcapsules. For WPI microcapsules, it is noticeable that the peaks at 1645 cm^−1^, 1539 cm^−1^ and 1240 cm^−1^ were related to the amide I (C=O stretching), amide II (N-H bending) and amide III (C-N stretching and N-H deformation) of WPI, respectively [36]. For OSA-starch microcapsules, the peak at 3000–3500 cm^−1^ was related to the O-H stretching of hydroxyl groups. The peaks at 1725 and 1572 cm^−1^ were observed for the stretching vibration of C=O and asymmetric stretching of COO^–^, respectively [37]. The FTIR spectrum of WPI-OSA-starch microcapsules was the combination of the FTIR spectrum of WPI microcapsules and OSA-starch microcapsules, although the location of the peaks was slightly changed, consistent with the report of Naderi et al. [38] and Zhang et al. [39]. In addition, the peaks shifted from 1645 cm^−1^ and 1539 cm^−1^ in the WPI microcapsules to 1650 cm^−1^ and 1544 cm^−1^ in the WPI-OSA-starch microcapsules, respectively, similar to the report of Naderi et al. [38] and Zhang et al. [39]. Furthermore, the peaks of OSA starch at 1725 cm^−1^ and 1571 cm^−1^ disappeared in the WPI-OSA-starch microcapsules, similar to the report of Zhao et al. [28]. The above changes confirmed the electrostatic attraction between the carboxyl groups (-COO^−^) of OSA starch and the amine groups (-NH^3+^) of WPI.

### 2.3. Microscopic Observations of Microcapsules Containing Probiotics

**Figure 3:**
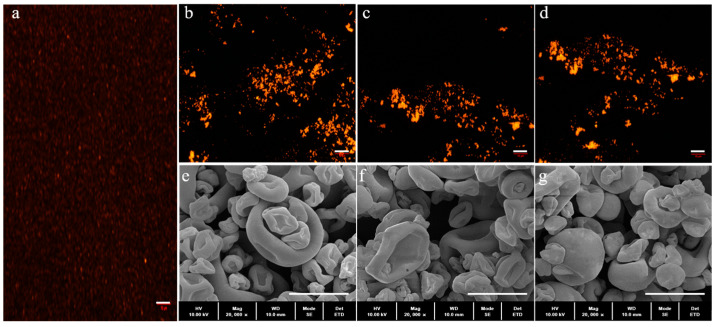
Fluorescence microscopy image of (**a**) aqueous suspension, (**b**) WPI microcapsules, (**c**) OSA-starch microcapsules, (**d**) WPI-OSA-starch microcapsules containing probiotic bacteria labelled with RBITC and SEM image of (**e**) WPI microcapsules, (**f**) OSA-starch microcapsules, (**g**) WPI-OSA-starch microcapsules. The bars in the fluorescence images and SEM images represent 10 μm and 5 μm, respectively.

Fluorescence microscopy was used to confirm the presence of probiotic bacteria in the microcapsules. As shown in Figure 3a, after labelling with RBITC, free probiotic bacteria can be seen in red under the fluorescence microscope, similar to the report by Peñalva et al. [23]. Although there is some overlap, the encapsulation of probiotic bacteria in WPI, OSA-starch and WPI-OSA-starch microcapsules can still be confirmed by the presence of red fluorescence in all microcapsules (Figure 3b–d).

The microstructure of the spray-dried microcapsules containing probiotics is also shown in Figure 3e–g. The sizes of the three microcapsules showed almost no difference. Some hollows and wrinkles were observed in both WPI microcapsules and OSA-starch microcapsules, which may result from the denaturation of WPI and the shrinkage of the OSA starch, respectively, during the spray-drying process [13,20]. The combination of WPI and OSA starch produced the microcapsules with compact structures, smooth surfaces and almost no wrinkles, which can be attributed to the electrostatic attraction between WPI and OSA starch, preventing the shrinkage of OSA starch during spray drying [20]. In addition, almost no probiotic bacteria were visible on the surface of all spray-dried microcapsules, suggesting that the probiotic bacteria were embedded in the coacervate network.

### 2.4. Probiotic Viability, Water Content and Water Activity of Spray-Dried Microcapsules

The viability of *Lactobacillus acidophilus* in different microcapsules after spray drying is shown in Table 1. The viability of microencapsulated probiotic bacteria was 91.32 ± 1.87% and 70.75 ± 2.05% in WPI microcapsules and OSA-starch microcapsules, respectively. The low viability of probiotics in OSA-starch microcapsules after spray drying was also reported by Cruz-Benítez et al. [19], and could be attributed to the poor solubility and poor film-forming property of OSA starch, resulting in uneven film formation at the beginning of the drying process and shrunken and wrinkled surfaces of the spray-dried microcapsules [19,20].

WPI-OSA-starch microcapsules exhibited a significantly (*p* < 0.05) higher viability (95.94 ± 1.64%) than either WPI microcapsules or OSA-starch microcapsules, indicating that the combination of WPI and OSA starch would enhance the protective effect for probiotics during the process of spray drying. The high viability of probiotic bacteria in the spray-dried WPI-OSA-starch microcapsules produced here may also be due to the smooth surfaces and the compact structures of the microcapsules [1,40]. In addition, the hydroxyl groups of OSA starch could form hydrogen bonds with the cell membrane of probiotics, effectively reducing heat damage and protecting the cell membrane from degeneration during spray drying [9]. Similarly, significantly (*p* < 0.05) higher probiotic viability after spray drying was also found in soy-protein-isolate–peach-gum-polysaccharide, soy-protein-isolate–pectin, WPI–*Momordica charantia*-polysaccharide microcapsules than that in protein/polysaccharide-only microcapsules and free bacteria [9,10,41]. On the other hand, decreased encapsulation viability was reported when shellac was incorporated into WPI-based microcapsules, which could be attributed to the excessive hydrophobicity of shellac (the contact angle of shellac powder of 152.3°) [13,42].

As also shown in Table 1, the water content of all probiotic microcapsules was not more than 7%, which is in line with the standard acceptance values for spray-dried products [13]. The water content of the WPI-OSA-starch microcapsules was similar to that of the WPI microcapsules, but lower than that of the OSA-starch microcapsules. Similar results would also be reported that the water content of shellac microcapsules was significantly (*p* < 0.005) higher than that of WPI–shellac microcapsules and WPI microcapsules due to the low drying rate of shellac microcapsules, despite the strong hydrophobicity of shellac [13]. The a_w_ values were consistent with the water content, and a higher a_w_ (0.247 ± 0.004) was found in the OSA starch microcapsules than in the others.

### 2.5. Viability of Microencapsulated Probiotics under Different Conditions

#### 2.5.1. SGI Digestion

**Figure 4:**
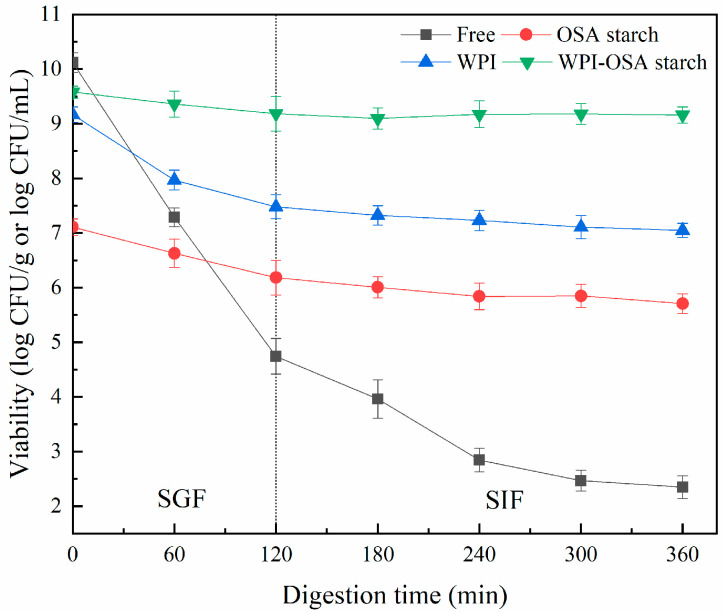
Viability of free and microencapsulated *Lactobacillus acidophilus* during simulated gastric (0–120 min) and intestinal (120–360 min) digestion.

As shown in Figure 4, the initial number of free *Lactobacillus acidophilus* was 10.12 ± 0.18 log CFU/mL, which decreased dramatically after SGI digestion, with viability of 4.75 ± 0.32 and 2.47 ± 0.19 log CFU/mL after digestion in SGF and SIF, respectively. After microencapsulation, the viability of *Lactobacillus acidophilus* after SGI digestion was significantly (*p* < 0.05) improved. Among all the microcapsules, the WPI-OSA-starch microcapsules showed the highest viability (9.16 ± 0.15 log CFU/g) after SGI digestion.

The significant (*p* < 0.05) increased viability of *Lactobacillus acidophilus* in WPI-OSA-starch microcapsules during SGI digestion could be mainly attributed to the smooth surface and the compact structure of the microcapsules, as well as the relatively high hydrophobicity of OSA starch, which were beneficial in reducing the hygroscopicity, wettability and solubility of the microcapsules and maintaining the integrity of the microcapsules during SGI digestion, thereby preventing the diffusion of hydrogen ions (H^+^), digestive enzymes, bile salts, etc., into the microcapsules [1,43,44]. In addition, the minimal damage to the probiotic bacteria in WPI-OSA microcapsules during spray drying, the ability of WPI to create a buffered microenvironment around probiotics and the resistance of OSA starch to digestion may also contribute to the improved viability of *Lactobacillus acidophilus* during SGI digestion after microencapsulation [9,18,44].

Similarly, significantly (*p* < 0.05) higher probiotic viability after SGI digestion was also found in soy-protein-isolate–peach-gum-polysaccharide, soy-protein-isolate–pectin, sodium-caseinate–gum-ghatti and sodium-caseinate–gum-arabic-based microcapsules than that in protein/polysaccharide-only microcapsules and free bacteria [9,10,45]. However, sodium caseinate–maltodextrin and sodium caseinate–pullulan microcapsules were unable to protect bacterial bacteria in SGF due to the extreme solubility of maltodextrin and pullulan in aqueous media, resulting in rapid dissolution of the microparticles [45].

#### 2.5.2. Heat Treatment

**Figure 5:**
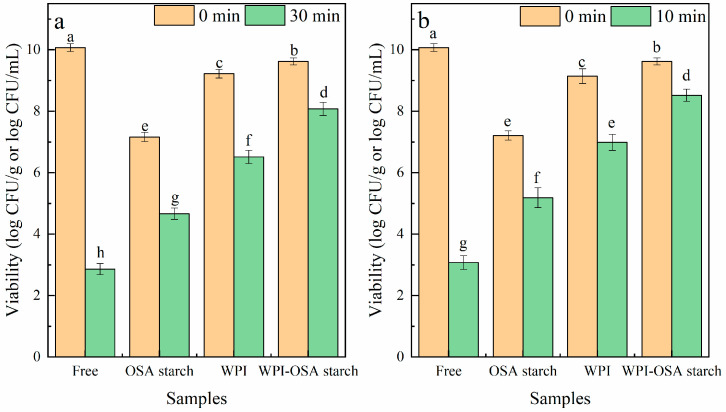
Viability of free and microencapsulated *Lactobacillus acidophilus* after thermal treatment of (**a**) 65 °C/30 min and (**b**) 75 °C/10 min. Significant differences (*p* < 0.05) are indicated by different lowercase letters.

The influences of heat treatments on the viability of *Lactobacillus acidophilus* are shown in Figure 5. The viability of free *Lactobacillus acidophilus* rapidly decreased from 10.07 ± 0.13 log CFU/mL to 2.86 ± 0.18 and 3.08 ± 0.23 log CFU/mL after heat treatment of 65 °C/30 min and 75 °C/10 min, respectively. After microencapsulation, the thermal stability of *Lactobacillus acidophilus* was significantly (*p* < 0.05) improved. The viability of microencapsulated *Lactobacillus acidophilus* in WPI-OSA-starch microcapsules was found to be 8.07 ± 0.25 log CFU/g and 8.52 ± 0.19 log CFU/g after thermal treatment of 65 °C/30 min and 75 °C/10 min, respectively, significantly (*p* < 0.05) higher than that in WPI microcapsules and OSA-starch microcapsules. The reason for this could be mainly attributed to the improved sealing properties of the microcapsules due to their compact structure and smooth surface, which slows down the heat diffusion.

Similar results were also reported previously—the viability loss of probiotic bacteria in soy-protein-isolate–pectin microcapsules was only 2.55 log CFU/g, which was lower than that in SPI microcapsules (3.1 log CFU/g) after thermal treatment (75 °C/10 min) [9]. However, as compared to soy-protein-isolate microcapsules, the combination of peach gum polysaccharide with soy-protein isolate reduced the viability of probiotic bacteria during the heat treatment due to the good solubility of peach-gum polysaccharide in aqueous solution, which accelerated the dissolution of the microcapsules [10]. These results suggest that the physicochemical properties of the polysaccharide are very important with respect to improving the structural compactness and thermal stability of the microcapsules.

#### 2.5.3. Storage

**Figure 6:**
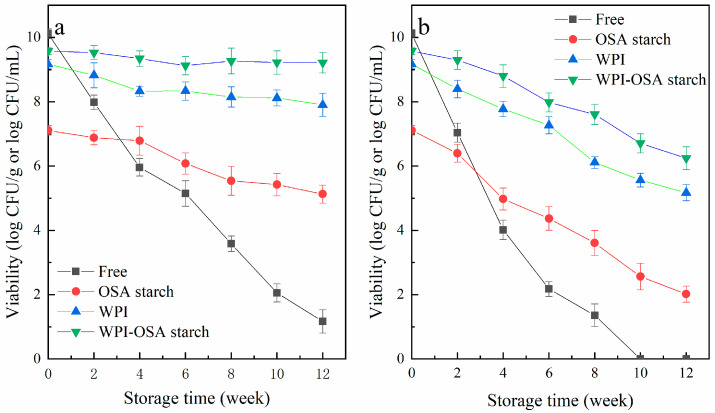
Viability of free and microencapsulated *Lactobacillus acidophilus* during storage at (**a**) 4 °C and (**b**) 25 °C.

As shown in Figure 6, the stability of the different microcapsules containing probiotics was evaluated during storage at temperatures of 4 °C and 25 °C. At 4 °C, the viability of free bacteria was decreased from 10.12 ± 0.15 log CFU/mL to 5.96 ± 0.27, 3.58 ± 0.24 and 1.17 ± 0.36 log CFU/mL, after storage for 4 weeks, 8 weeks and 12 weeks, respectively. At 25 °C, no free *Lactobacillus acidophilus* survived after 10 weeks of storage. The storage stability of *Lactobacillus acidophilus* was significantly (*p* < 0.05) improved by encapsulation, especially in WPI-OSA-starch microcapsules. Bacterial vitality was maintained at 9.22 ± 0.31 and 6.25 ± 0.36 log CFU/g after 12 weeks of storage at 4 °C and 25 °C, respectively. Thus, it can be concluded that WPI-OSA-starch microcapsules can effectively protect *Lactobacillus acidophilus* during storage, mainly due to the smoother surface and the more compact structure of the microcapsules, as well as the relatively high hydrophobicity of OSA starch, which retards the diffusion of oxygen, moisture and other substances into the microcapsules during storage [1,43]. Similarly, the significantly (*p* < 0.05) higher storage stability of probiotics in sodium-caseinate-gum-ghatti, sodium-caseinate–gum-arabic, soy-protein-isolate–peach-gum-polysaccharide and soy-protein-isolate–pectin microcapsules than those in protein/polysaccharide-only microcapsules and free bacteria has also been reported [9,10,45].

## 3. Materials and Methods

### 3.1. Material

*Lactobacillus acidophilus* 11073 was obtained from Gaofuji Biological Technology Co., Ltd. (Chengdu, China). Rice starch (amylose content of 21.54 ± 0.85%) was obtained from Haixi Biological Technology Co., Ltd. (Guangzhou, China). WPI was obtained from Haofa Biological Technology Co., Ltd. (Zhengzhou, China). De Man, Rogosa Sharp (MRS) agar and broth were obtained from Aoboxing Biological Technology Co., Ltd. (Beijing, China). OSA, pepsin (P7012), pancreatin (P7545), amyloglucosidase (A7095), porcine-bile extract (B8613) and rhodamine B isothiocyanate (RBITC) were obtained from Sigma-Aldrich (St. Louis, MO, USA). Other analytical pure chemical reagents were obtained from Cologne Chemical Co. (Chengdu, China).

### 3.2. Synthesis of OSA Starch

OSA starch was synthesised following a previously reported process [46]. Briefly, 100 g of rice starch was mixed with 400 mL of distilled water. After adjusting the pH of the mixture to 8.5 with 1 M sodium hydroxide (NaOH) solution, 3 g of OSA was slowly added while keeping the pH at 8.5. After 6 h of reaction at 25 °C, the pH was lowered to 7.0 with 1 M hydrochloric acid (HCl) solution (1 M). The precipitate obtained via centrifugation was washed three times with distilled water and then once with acetone. The OSA starch obtained was dried to the initial moisture content of starch, ground to powder and then passed through a 100-mesh sieve.

### 3.3. Complex Coacervation Formation and Characterisation

#### 3.3.1. Preparation of the WPI-OSA-Starch Solution

The preparation of WPI solution (1%, *w*/*v*) was performed by mixing the powder of WPI with aqua destillata and slowly stirring until complete hydration was achieved. The OSA-starch solution (1%, *w*/*v*) was prepared by mixing the powder of OSA starch with aqua destillata, followed by gelatinisation at 95 °C for 20 min. As the retrogradation usually occurred in gelatinised starch, the fresh OSA starch solution had to be prepared for each experiment and used immediately after preparation.

#### 3.3.2. Turbidity Measurement

Turbidity measurements were performed at different ratios (WPI:OSA starch of 0:1, 0.5:1, 1:1, 2:1, 3:1 and 1:0) with a pH range of 2.0–6.5 using a UV-vis spectrophotometer (SDPTOP UV-2400, Shanghai, China) at 600 nm. In order to adapt the microencapsulation and keep the turbidity values in an appropriate range, measurements were made at a relatively high concentration (total biopolymer concentration of 0.2%, *w*/*v*), using the glass cells with an optical path of 0.5 cm [47]. The optimum pH (pHopt) was defined as the pH at which maximum turbidity was achieved [48].

#### 3.3.3. Zeta Potential Analysis

The zeta potential of the solution of WPI, OSA starch and the mixture of them at different ratios (WPI:OSA starch of 0.5:1, 1:1, 2:1 and 3:1) was determined as a function of pH (3.0–6.5, adjusted with an HCl solution) using a zetasizer (Nano-ZS, Malvern Instruments, Ltd., Worcestershire, UK). The pH at which the zeta potential reaches 0 was defined to be the isoelectric point (IEP) [48].

#### 3.3.4. Yield Measurement of Complex Coacervates

In order to study the adaptability of the process in the field of microencapsulation, the yields of WPI-OSA-starch-complex coacervates with different ratios (WPI:OSA starch of 0.5:1, 1:1, 2:1 and 3:1) and different total polymer concentrations (1% and 5%, *w*/*v*) were determined following the approach described by Carpentier et al. [48] and Chen et al. [47]. Briefly, the pH of each mixed solution of WPI-OSA starch was adjusted to its pHopt. When phase separation was evident, the liquid complex coacervates were collected via centrifugation (9000× *g*, 20 min) and then dehydrated at 105 °C to constant mass. The yield of each complex coacervate was calculated according to Equation (1):

Yield (%) = W_c_/W_t_ × 100 (1)

In this equation, W_c_ is the dry weight of the complex coacervates (g) and W_t_ is the sum of the dry weight of WPI and OSA starch used for the complex coacervation (g).

### 3.4. Microencapsulation of Probiotic Bacteria

Glycerol-preserved *Lactobacillus acidophilus* was incubated in an MRS culture medium at 37 °C for 24 h to prepare *Lactobacillus acidophilus* inoculum, which was then transferred to MRS culture medium and incubated for a further 24 h. The bacterial suspension was centrifuged (6000× *g*/5 min) and the pellet was washed twice and redispersed in sterile normal saline solution to give a final concentration of approximately 10^10^ CFU/mL.

Microcapsules were prepared as follows: (1) prepare WPI, OSA starch and WPI-OSA-starch-mixed solution, the ratio and the total concentration were determined in Section 2.3; (2) keep the solution at 4 °C for 12 h to ensure adequate hydration; (2) add probiotic bacteria solution to the above solutions to about 10^10^ CFU/g materials and stir continuously for 30 min; (3) adjust the pH to the pHopt determined in Section 2.3 and keep the above solutions at 4 °C for 12 h to ensure the completion of the complex coacervation; (4) dehydrate the complex coacervates containing the probiotic bacteria using a spray dryer (EYELA SD-1000, Tokyo Rikakikai, Ltd., Tokyo, Japan) with the following parameters: inlet/outlet temperature 120 °C/70 °C; gas flow rate 30 m^3^/h.

### 3.5. Characterisation of the Microcapsules

#### 3.5.1. FTIR Spectroscopy

The FTIR spectrum of the different microcapsules containing probiotics was measured using an FTIR spectrometer (Nicolet iS10, Thermo Fisher Scientific, Ltd., Waltham, MA, USA). Briefly, 2 mg of microcapsule powder and 98 mg of KBr were blended and ground to a fine powder using an agate mortar. The ground powder was then pressed into transparent flakes using a tablet press. The range of the wave number for measurement was set to 400–4000 cm^−1^. The spectral data were obtained via 32 scans with a resolution of 1 cm^−1^.

#### 3.5.2. Fluorescence Microscopy

*Lactobacillus acidophilus* were labelled with RBITC according to the approach reported by Peñalva et al. [23]. Different microcapsules containing RBITC-labelled bacteria were prepared as described in Section 2.4 and analysed via an inverted fluorescence microscope (Nikon TS100, Tokyo, Japan).

#### 3.5.3. Microstructure Observation

A scanning electron microscope (SEM) (SU8010, Hitachi Science Systems, Ltd., Tokyo, Japan) was used to measure the microstructure of the different microcapsules containing probiotics. After, they were placed on the adhesive carbon discs and coated with a gold layer. Images of each sample were obtained at 10.0 kV accelerating potential and 20,000× magnification.

#### 3.5.4. Water Content and Water Activity

The water content of the different microcapsules containing probiotics was measured by removing the water at 105 °C to constant mass. The water-activity meter (LabMas-terav., Lachen, Switzerland) was used to analyse the water activity (a_w_) of different microcapsules containing probiotics at 25 °C.

### 3.6. Determination of Microencapsulation Viability

Following the approach reported by Zhao et al. [49], microcapsules (dry weight) were dispersed in sterile normal saline solution at a ratio of 1:99 (*w*:*v*), kept at 37 °C for 60 min and then vortexed to homo-dispersion. The suspension was then serially diluted and counted via the standard plate count (SPC) method using MRS agar. Incubation of the plates was performed at 37 °C for 2 days.

### 3.7. Viability under Different Treatments

#### 3.7.1. SGI Digestion

The viability of free and microencapsulated probiotic bacteria after SGI digestion was determined following the approach described by Mao et al. [50], with some modifications. The sterilised sodium chloride (NaCl) solution (0.2%, *w*/*v*, pH 2.0) containing pepsin (0.3%, *w*/*v*) was prepared and used as the simulated gastric fluid (SGF). The NaCl solution (0.2%, *w*/*v*, pH 8.0) containing pancreatin (0.1%, *w*/*v*) and bile salts (0.3%, *w*/*v*) was prepared and used as the simulated intestinal fluid (SIF). For SGI digestion, microcapsules (0.1 g) or free (0.1 mL) probiotic bacteria were added to 9.9 mL SGF and kept at 37 °C for 120 min. At 60 and 120 min, 0.1 mL of SGF was taken from each sample, serially diluted and counted via the SPC method. After simulated gastric digestion, the rest of SGF was moved to the SIF and the probiotic bacteria were counted at 60, 120, 180, 240, 300 and 360 min as previously described.

#### 3.7.2. Thermal Treatment

To measure the thermal stability, microcapsules (1 g) or free (1 mL) probiotic bacteria were mixed with 9 mL sterile normal saline solution, and then incubated in a thermostat water bath (65 °C/30 min or 75 °C/10 min) followed by cooling with ice. The number of viable bacteria after thermal treatment was then determined.

#### 3.7.3. Storage

To determine the storage stability, microcapsules (1 g) or free (1 mL) probiotic bacteria were preserved in sealed tubes at 4 °C/25 °C for 12 weeks. Each week, 0.1 mL of free bacteria or 0.1 g of microcapsules were taken from each sample and counted as previously described.

### 3.8. Statistical Analysis

Each measurement was performed three times and the data were recorded as the mean ± standard deviation. Statistical differences between samples were analysed using SPSS software, with one-way analysis of variance (ANOVA) and Tukey’s test (*p* < 0.05).

## 4. Conclusions

In this study, complex coacervates of WPI and OSA starch were prepared for microencapsulation of *Lactobacillus acidophilus*. The optimum mixing ratio and pH for the preparation of the WPI-OSA-starch-complex coacervate were found to be 2:1 and 4.0, respectively. The occurrence of an electrostatic interaction between WPI and OSA starch in microcapsules was confirmed via FTIR. The spray-dried WPI-OSA starch microcapsules exhibited more compact structures and smoother surfaces than WPI-OSA starch-only microcapsules. Microencapsulation via the complex coacervation of WPI-OSA starch was found to be effective in improving the viability of *Lactobacillus acidophilus* during spray drying, SGI digestion, thermal treatment and long-term storage. Taken together, these results demonstrate that WPI-OSA-starch-complex coacervates are promising wall materials for probiotic encapsulation.

## Figures and Tables

**Table 1:** Probiotic viability, water content and water activity of spray-dried microcapsules.

Samples	Log N_0_ (log CFU/g)	Log N_t_ (log CFU/g)	Viability Log N_t_/Log N_0_ (%)	Water Content (%)	Water Activity
WPI	10.03 ± 0.11 ^a^	9.16 ± 0.17 ^b^	91.32 ± 1.87 ^b^	4.43 ± 0.36 ^b^	0.205 ± 0.009 ^b^
OSA starch	10.05 ± 0.14 ^a^	7.11 ± 0.19 ^c^	70.75 ± 2.05 ^c^	5.26 ± 0.15 ^a^	0.247 ± 0.004 ^a^
WPI-OSA starch	10.09 ± 0.12 ^a^	9.68 ± 0.13 ^a^	95.94 ± 1.64 ^a^	4.68 ± 0.28 ^b^	0.208 ± 0.005 ^b^

^a^ N_0_ is the viability of *Lactobacillus acidophilus* used for microencapsulation; N_t_ is the viability of *Lactobacillus acidophilus* after spray drying. ^b^ Different lowercase letters in the same column mean significant differences (*p* < 0.05).

## Data Availability

Data is contained within the article.

## Sample Availability

Not available.
